# Expression of lncRNA *MIR222HG* co-transcribed from the *miR-221/222* gene promoter facilitates the development of castration-resistant prostate cancer

**DOI:** 10.1038/s41389-018-0039-5

**Published:** 2018-03-13

**Authors:** Tong Sun, Shin-Yi Du, Joshua Armenia, Fangfang Qu, Jingyu Fan, Xiaodong Wang, Teng Fei, Kazumasa Komura, Shirley X. Liu, Gwo-Shu Mary Lee, Philip W Kantoff

**Affiliations:** 1 Department of Medical Oncology, Dana-Farber Cancer Institute and Harvard Medical School, 450 Brookline Ave, Boston, MA 02215 USA; 2 Department of Medicine, Memorial Sloan Kettering Cancer Center, 1275 York Avenue, New York, NY 10065 USA; 3 Human Oncology and Pathogenesis Program, Memorial Sloan Kettering Cancer Center, New York, NY USA; 4 Center for Functional Cancer Epigenetics, Dana-Farber Cancer Institute, Boston, MA 02215 USA

## Abstract

Mechanisms by which non-coding RNAs contribute to the progression of hormone-sensitive prostate cancer (PCa) (HSPC) to castration-resistant PCa (CRPC) remain largely unknown. We previously showed that *microRNA-221/222* is up-regulated in CRPC and plays a critical role in modulating androgen receptor function during CRPC development. With further investigation, we characterized a putative promoter region located 23.3 kb upstream of the *miR-221/222* gene, and this promoter is differentially activated in CRPC LNCaP-Abl cells, leading to the up-regulation of *miR-221/222*. Upon promoter activation, a set of polyadenylated long non-coding RNA (lncRNA) *MIR222HGs* was transcribed from this promoter region. Over-expression of these *MIR222HGs* increased androgen-independent cell growth and repressed the expression of androgen receptor-regulated dihydrotestosterone (DHT)-induced *KLK3*, *TMPRSS2*, and *FKBP5* in HSPC LNCaP cells, hallmarks of the CRPC phenotype. Clinically, increased expression of *MIR222HG* is associated with PCa progression to CRPC. In primary tumors, expression levels of *MIR222HG* and *miR-221/222* inversely correlate with Gleason score and androgen receptor (AR) pathway activity. Interestingly, *MIR222HG* is Argonaute 2-bound and its expression is Dicer 1-dependent, suggesting its functional association with the RNA-induced silencing complex. Further studies led to the hypothesis that *MIR222HG* may potentially affect miR-mediated expression silencing, subsequently leading to AR reprogramming. Our study highlights an essential role of a non-coding RNA in CRPC development and that differential activation of a single promoter can up-regulate two different types of non-coding RNAs, *miR-221/222* and lncRNA *MIR222HG*, in CRPC. Additionally, this study reveals a novel function of lncRNAs as a modulator of Argonaute-mediated RNA-induced silencing complex.

## Introduction

Prostate cancer (PCa) is the most common non-cutaneous malignancy diagnosed in American men and the second leading cause of cancer mortality in men^1^. Androgens and the androgen receptor (AR) play crucial roles in PCa development and progression. Androgen deprivation therapy (ADT) remains a key treatment for advanced PCa. Most hormone-sensitive PCa patients initially respond to ADT; however, ultimately most patients develop resistance to ADT and progress to the lethal castration-resistant PCa (CRPC)^2,3^. PCa cells utilize a variety of AR-dependent and AR-independent pathways to survive in an androgen-depleted environment during CRPC progression. Many studies have shown that CRPC is frequently characterized by altered AR expression and persistent AR signaling activated by residual androgens^4–6^. However, our understanding of the mechanisms underlying CRPC development and progression remains limited.

Non-coding RNAs (ncRNAs) are major components of the eukaryotic transcriptome^7^. Based on their size range, ncRNAs are classified into small (<200 nucleotides) and long ncRNAs (lncRNAs; >200 nucleotides). These ncRNAs play important regulatory roles in diverse biological processes including cancer development and progression^8^. Among the different classes of ncRNAs, microRNAs (miRs) are relatively conserved groups of small ncRNAs ranging between 19 and 25 nucleotides that negatively regulate gene expression. Unlike small ncRNAs, lncRNAs are less conserved among different species and are usually expressed at relatively low levels. The major functions of identified lncRNAs, thus far, include (a) regulation of gene expression, both transcriptionally or post-transcriptionally, (b) facilitation of the formation of functional transcriptional complexes at promoter or enhancer sites by induction of chromatin structural changes, and (c) recruitment of various effector molecules or direct interaction with other RNAs^9,10^. Increasing numbers of cancer-related lncRNAs have been identified;^11^ however, many of their biological mechanisms leading to cancer development and progression remain unknown. It has been shown that lncRNAs, *PCA3* and *PCAT-1*, are up-regulated in PCa and that the lncRNA, *SChLAP1*, is associated with aggressive PCa^12–14^. We recently demonstrated that lncRNAs could work through a “sponge” mechanism competing for miRs that regulate phosphatase and tensin homolog (PTEN) expression, an important tumor suppressor gene in PCa progression^15^. We anticipated that additional novel functional mechanisms of ncRNAs may be involved in the development of CRPC.

Previously, we found that elevated expression of *miR-221/222* is associated with human CRPC. In vitro, up-regulation of *miR-221/222* expression conferred androgen-independent cell growth, reduced the transcription of a subset of androgen-responsive genes, and promoted the activation of epithelial–mesenchymal transition/tumor metastasis pathways^16,17^. Thus, up-regulation of *miR-221/222* potentially leads to reprogramming of AR signaling, which in turn may mediate the transition to the CRPC phenotype. We continued our investigation of the structure of the *miR-221/222* locus to help understand the potential mechanisms involved in the regulation of *miR-221/222* expression in CRPC.

We present here the characterization of *miR-221/222* locus and the role of the promoter co-transcribed *MIR222HG* lncRNA*s* in the development of CRPC phenotype. Our study suggests that up-regulation of *miR-221/222* and lncRNA *MIR222HG* expression in CRPC are most likely driven by the activation of the same promoter and both of them are involved in the progression from hormone-sensitive prostate cancer (HSPC) to CRPC.

## Results

### LncRNA *MIR222HGs* are coordinately transcribed from the *miR-221/222* gene promoter

**Fig. 1:**
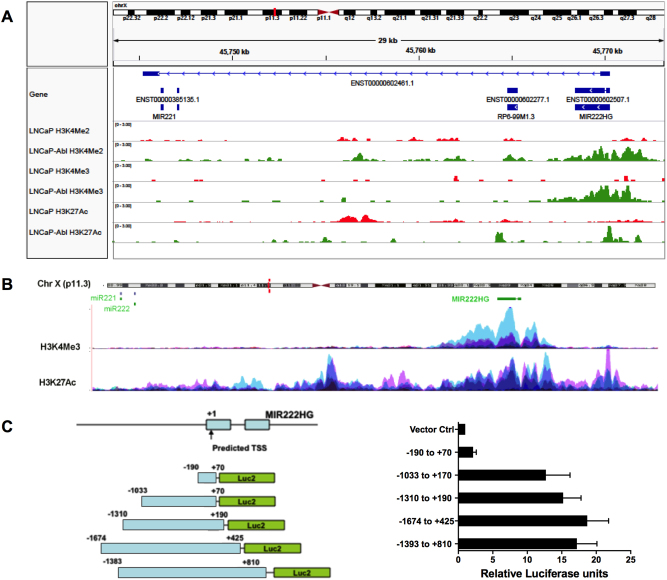
Identification of a promoter driving the transcription of the *miR-221/222* locus. **a** H3K4Me3, H3K4Me2, and H3K27Ac marks in LNCaP and LNCaP-Abl. Top panel, the diagram of *miR-221/222* gene and MIR222HG loci in Chr. Xp11. 3. All CHIP-seq data were documented in www.cistrome.org. **b** Upper panel, H3K4Me3 marks on seven cell lines from ENCODE hg19; lower panel, H3K27Ac marks on seven non-PCa cell lines (GM12878, Hi-hESC, HSMM, HUVEC, K562, NHEK, and NHLF) from ENCODE hg19 (www.genome.ucsc.edu). **c** Characterization of a promoter region at the 5′ region of the *MIR222HG* gene. Left panel, fragments of the promoter region were cloned into the upstream of a luciferase reporter gene. Right panel, promoter luciferase reporter assays in LNCaP-Abl cells. Relative luciferase activities were calculated in relative to the vector control (*Renilla* luciferase vector without a promoter) whose activity was arbitrarily set as 1

We previously showed that *miR-221/222* is frequently over-expressed in CRPC tumors, participating in the CRPC development. *MiR-221/222* is expressed >10-fold higher in the androgen-independent LNCaP-Abl cell line compared to that of the androgen-dependent LNCaP^16,17^. LNCaP-Abl cell line was established by a long period of adapting growth of LNCaP in medium containing charcoal-stripped fetal bovine serum (CFBS). The whole chromosome AR binding sites in LNCaP and LNCaP-Abl have been mapped and the dynamics of AR transcription complex loading on the regulatory regions are well characterized^18^. To address the mechanisms underlying the transcriptional regulation of *miR-221/222* in PCa cells, we identified the potential regulatory region of the *miR-221/222* gene locus by comparing histone modification marks in LNCaP and LNCaP-Abl cell lines with further confirmation using ENCODE hg19 histone modification data (Fig. 1).

We compared the H3K4Me2 (define transcription factor binding sites), H3K4Me3 (highly enriched at active promoters near transcription start sites, TSS) and H3K27Ac (associated with a higher transcriptional activation that defines an active enhancer mark) chromatin immunoprecipitation sequencing (ChIP-seq) data between LNCaP and LNCaP-Abl^18^. We noticed that a cluster of high-density active transcription histone marks H3K4Me3 and H3K4Me2 was located −23.3 kb upstream of the *miR-221/222* gene in LNCaP-Abl, but not in LNCaP (Fig. 1a). Consistently, the H3K27Ac mark, associated with transcriptional initiation and open chromatins, was also detected in the same location in LNCaP-Abl, but not in LNCaP (Fig. 1a). With further comparison of ENCODE data, we confirmed that the dense H3K4Me3 marks, and a wider H3K27Ac mark at −23.3 kb upstream of the *miR-221/222* gene were also present in many other non-PCa cell lines (Fig. 1b; https://genome.ucsc.edu/). Additionally, a DNAase I hypersensitivity cluster site (from 125 cell types) also co-localized in the same position, indicating possible transcriptional activity (https://genome.ucsc.edu/)^19^. Taken together, these data strongly suggested that a promoter region was located at −23.3 kb upstream of the *miR-221/222* gene, and, most likely, the activation of this upstream promoter leads to the differential up-regulation of *miR-221/222* expression in LNCaP-Abl.

We further analyzed the nucleotide sequence of this putative promoter of *miR-221/222* gene to identify TSS. By employing the Promoter 2.0 Prediction Server^20^, an RNA polymerase II TSS was predicted with a 1.046 score, indicating that a promoter was highly likely to be in this region (Fig. S1). Consistently, the predicted promoter region overlaps with H3K4me3 and H3K4me2 histone modification marks. By sequential deletion analysis of promoter activity, the essential domain of this upstream putative *miR-221/222* promoter was defined (Fig. 1c). We found that the DNA fragment −1393 to +810 (+1 was the predicted TSS) and the −1674 to +425 fragment exhibited similar strong promoter activity. The promoter activity was slightly reduced in the reporter constructs containing promoter fragments −1310 to +190 and −1033 to +170 and was completely lost in the fragment −190 to +70. These data suggested that the core promoter could be located in the fragment between −1033 and −190. This was consistent with the transcriptional activities assayed by RNA-seq on nine cell lines from ENCODE demonstrating that baseline transcription events started ~23.3 kb upstream of the *miR-221/222* gene and proceeded to the *miR-221/222* gene^19^.

Interestingly, several transcripts, named *MIR222HGs*, derived from this *miR-221/222* promoter region were found in the ENCODE database (Fig. 1a). The sizes of the annotated *MIR222HGs* vary in size from ~1 to ~23 kb. A long precursor transcript spanning the entire 23.3 kb from the promoter region to the 3′ end of the *miR-221/222* gene was also noted. We hypothesized that this remote upstream promoter may drive transcription of the entire 23 kb locus and the precursor *miR-221/222* and *MIR222HGs* might be generated from the same primary transcript.

### *MIR222HGs* are spliced and polyadenylated lncRNAs

**Fig. 2:**
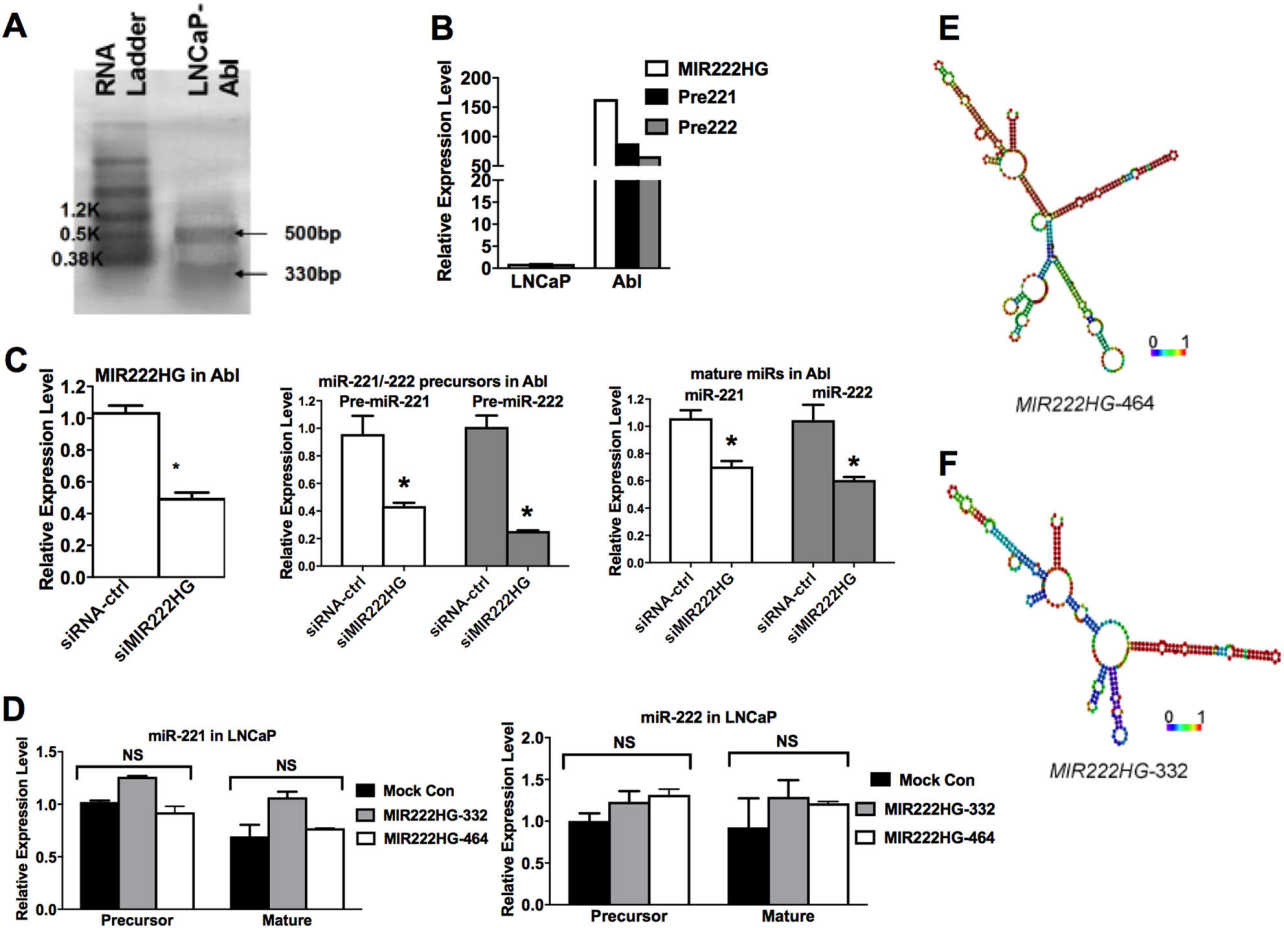
Characterization of *MIR222HG* lncRNA. **a** Northern blot of *MIR222HG* lncRNA in LNCaP-Abl cells. Poly(A)-tailed RNA from LNCaP-Abl was separated on a 1.5% denaturing agarose gel and was hybridized with DIG-labeled DNA probes. Two horizontal arrows indicate two different isoforms of *MIR222HG* lncRNA. **b** The expression levels of *MIR222HG* (white bars), *miR-221* precursors (black bars), and *miR-222* precursors (gray bars) in LNCaP and LNCaP-Abl cell lines. **c** Knocking down *MIR222HG* lncRNA reduced the expression levels of precursors and mature *miR-221/222*. Left panel, *MIR222HG* siRNA efficiency in LNCaP-Abl cells; middle panel, *miR-221/222* precursors in LNCaP-Abl cells after *MIR222HG* knocking down; right panel, mature *miR-221/222* expression in LNCaP-Abl after *MIR222HG* knocking down. **d** Over-expressing *MIR222HG* lncRNA had no impact on *miR-221/222* expression. Left panel, the level of precursor and mature miR-221 in LNCaP after over-expressing two different isoforms of *MIR222HG* in LNCaP cells; right panel, the level of precursor and mature *miR-222* in LNCaP after over-expressing two different isoforms of *MIR222HG*. **e**, **f** RNAfold secondary structure predictions of *MIR222HG*-464 and *MIR222HG*-332, with the minimum free energy as −108.7l and −82.7 kcal/mol, respectively. The color scale indicates high (red) to low (blue) probabilities of base pairing. RNA secondary structures and energies were predicted using RNAfold from the Vienna RNA package

We investigated whether *MIR222HG*-related transcripts are present in LNCaP-Abl, which exhibits active transcription at the 23 kb *miR-221/222* locus. We performed Northern blotting analysis of poly(A)+ RNA from LNCaP-Abl and identified two different length transcripts of ~330 and ~500 bp, respectively (Fig. 2a). Using a strand-specific reverse transcription-polymerase chain reaction (RT-PCR) assay, we found that these two different variants were transcribed in the same orientation as the *miR-221/222* gene. By 5′-rapid amplification of cDNA ends (5′RACE) and 3′RACE, we determined the sequence of the two *MIR222HG* isoforms of 332 and 464 bp, respectively (Fig. S1). Nucleotide sequence analysis indicated that the *MIR222HG*-332 bp isoform was spliced from the −464 bp isoform by removing one 132 bp intron. We examined the potential protein-coding capacity of *MIR222HG* sequences. Three potential open reading frames (ORFs), 37 to 80 amino acids in length, were predicted in each of the *MIR222HG* isoforms. These short ORFs lacked the Kozak sequence and had negative coding potential calculator (CPC) scores, implying that the RNAs were CPC classified as non-coding^21^. These results indicated that the 332 and 464 bp *MIR222HGs* identified in LNCaP-Abl are unlikely encoding functional peptides and they are spliced and polyadenylated lncRNAs.

The expression level of *MIR222HGs* in PCa cell lines was determined by strand-specific RT-PCR. Consistently with our H3K4me2 ChIP-seq data, we found *MIR222HGs*' expression was high in LNCaP-Abl, while only barely detectable in LNCaP (Fig. 2b). Since it is known that some promoter associated lncRNAs play important roles in the transcription regulation of genes driven by the same promoter, we further investigated the impact of expression of lncRNA *MIR222HGs* on *miR-221/222* expression. We found that knocking down *MIR222HGs* in LNCaP-Abl significantly down-regulated *miR-221/222* gene expression, especially at the *miR-221/222* precursor level (Fig. 2c). However, over-expressing the two *MIR222HGs* had no impact on the expression of *miR-221/222* (Fig. 2d). Taken together with the fact that the ENCODE data demonstrated the presence of a long transcript spanning from *MIR222HG* to the *miR-221/222* gene, we concluded that unlike other promoter associated lncRNAs, the *MIR222HG*s most likely were not involved in the transcriptional regulation of the *miR-221/222* locus. We surmised that *MIR222HG* and *miR-221/222* most likely originate from the same polycistronically transcribed primary transcript in LNCaP-Abl. Thus, knocking down the endogenous *MIR222HGs* subsequently led to knockdown of the long primary transcript from which *miR-221/222* was derived, resulting in the reduction of *miR-221/222*. Additional structural analysis using the RNAfold program^22^ indicated that *MIR222HG*-332 and *MIR222HG*-464 form stable stem-loop structures (Fig. 2e, f), suggesting that *MIR222HGs* potentially function as regulatory RNAs, which prompted us to further investigate their potential roles in PCa progression.

### *MIR222HGs* are involved in the CRPC phenotype in PCa cell lines

**Fig. 3:**
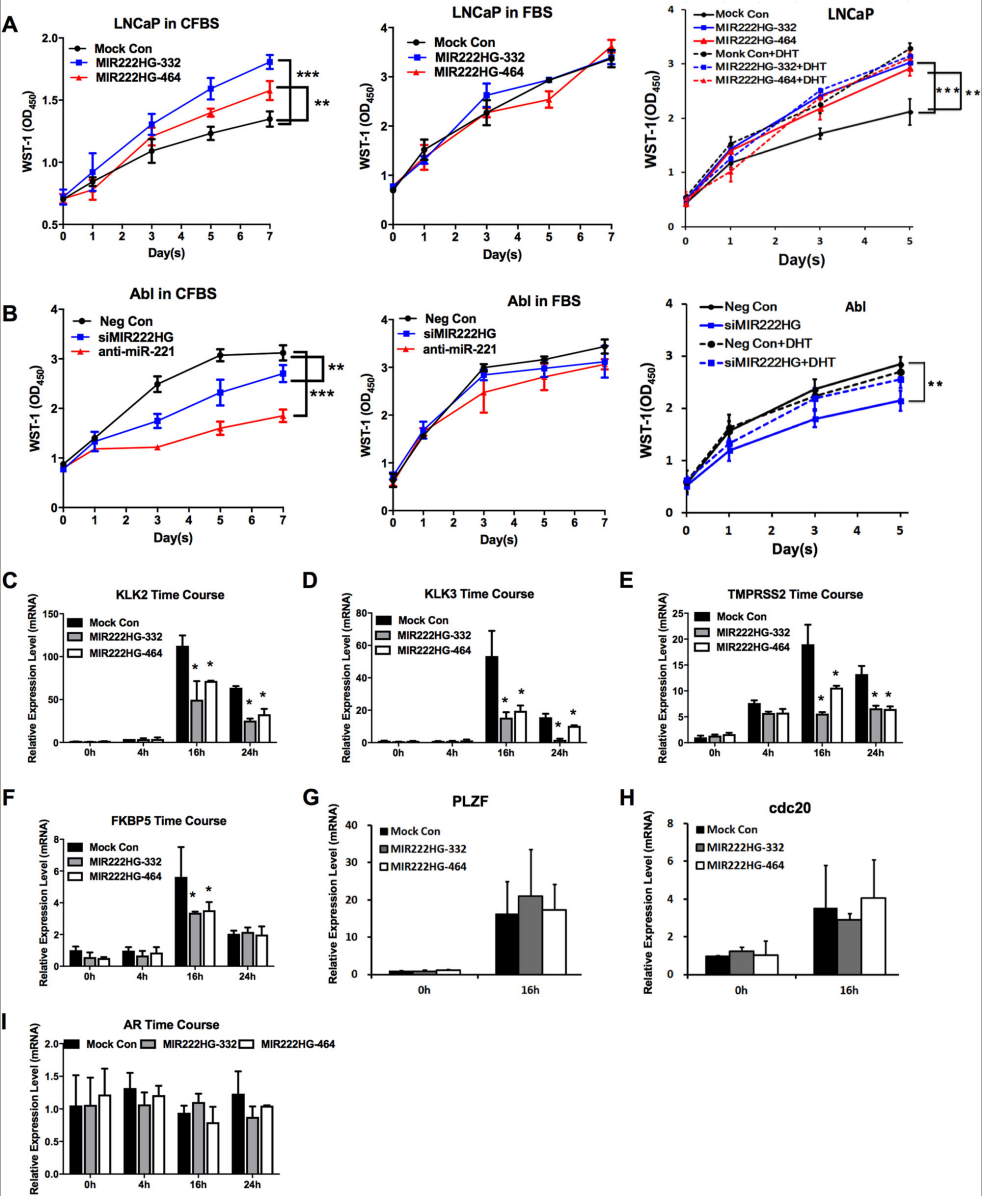
The impact of *MIR222HG* expression levels on CRPC characteristics. **a** Effect of *MIR222HG* expression level on the growth of LNCaP. LNCaP cells that were transfected with the empty vector (Mock Con, black lines), *MIR222HG*-332 bp isoform (blue lines) or 464 bp isoform (red lines) in hormone-free medium (CFBS, left panel), in regular medium (FBS, middle panel), or in CFBS plus 10 nM DHT (right panel; solid lines indicating CFBS only; dotted lines indicating CFBS + DHT). **b** Effect of *MIR222HG* expression level on the growth of LNCaP-Abl. LNCaP-Abl cells that were transfected with the negative siRNA control (black lines), anti-miR-221 (red lines), or *MIR222HG* siRNAs (blue lines) and kept in hormone-free medium (CFBS, left panel), in regular medium (FBS, middle panel), or in CFBS treated with 10 nM DHT(right panel; solid lines indicating CFBS only; dotted lines indicating CFBS + DHT). Cell growth was measured by the WST-1 assay. Triplicate experiments were performed for each set. The data represents mean ± S.D. (*n* = 3). *The WST-1 fold changes at day 7 after transfection with a *p *value <0.01 (**) or *p *value <0.001 (***) in one-way ANOVA. **c–h** The impact of *MIR222HG* expression level on the AR-mediated transcription in response to the DHT treatment. Quantitative analysis of the expression level of *KLK2* (**c**), *KLK3* (**d**), *TMPRSS2* (**e**), *FKBP5* (**f**), *PLZF* (**g**), and *cdc20* (**h**) in LNCaP upon DHT treatment. **i** The AR mRNA expression level in *MIR222HG*-over-expressing cells. The relative expression levels of AR and AR-mediated genes in each sample were normalized with the expression level of GAPDH. Values represent the fold differences relative to those in cells without any drug treatment or transfection (Mock), which were set as 1.0. *The fold changes of those transfected samples compared with their corresponding negative controls show a *p *value <0.05 in one-way ANOVA

To explore the potential biological function of lncRNA *MIR222HGs* in PCa cells, we evaluated the importance of *MIR222HGs*’ expression on the development or maintenance of CRPC, since their expression is higher in the CRPC LNCaP-Abl compared to that in the HSPC cell line LNCaP. We determined the impact of changing *MIR222HG* expression on growth efficiency of LNCaP and LNCaP-Abl in the presence or absence of androgen. Over-expressing either *MIR222HG-332* or *MIR222HG-464* in LNCaP did not alter cell growth either in the regular medium or the medium with CFBS and DHT, though interestingly it significantly increased the growth of LNCaP in androgen-free CFBS by ~50% at day 7 (Fig. 3a). The enhanced androgen-independent cell growth seen with over-expression of *MIR222HGs* was also observed in another androgen-dependent cell line, LAPC4 (Fig. S2). Since *MIR222HGs* are up-regulated in LNCaP-Abl, we examined the impact of knocking down *MIR222HG* on the growth efficiency of LNCaP-Abl. Knocking down *MIR222HG* significantly reduced cell growth of LNCaP-Abl in CFBS, but did not affect the growth of LNCaP-Abl in either CFBS with DHT or the regular medium (Fig. 3b). Similarly, knocking down *miR-221* expression significantly affected the growth rate of LNCaP-Abl in androgen-free medium, but not in medium containing androgen as shown in Fig. 3b and as previously reported^16,17^. These data suggested that expression of *MIR222HGs* promotes androgen-independent growth of PCa cells, a hallmark of CRPC.

We further investigated whether AR signaling may be compromised due to the expression of *MIR222HGs* in LNCaP leading to the development of CRPC phenotype, by examining the level of AR-mediated transcription in response to DHT treatment. The *MIR222HG* level in LNCaP-*MIR222HG*-over-expressing cells was confirmed by RT-PCR and a ~4-fold over-expression of *MIR222HG* was observed (Fig. S9). We examined the expression level of a series of AR-regulated genes including *KLK2*, kallikrein-related peptidase 2 gene; *KLK3*, kallikrein-related peptidase 3 gene (or PSA, a marker for PCa screening); *TMPRSS2*, transmembrane protease serine 2 gene; *FKBP5*, peptidyl-prolyl *cis–trans* isomerase (or FK506 binding protein 5) gene; *PLZF*, promyelocytic leukemia zinc finger protein, which is a putative tumor suppressor gene in PCa; and *cdc20*, cell division cycle 20 homolog. Over-expression of the two isoforms of *MIR222HG* in LNCaP significantly reduced the DHT-induced expression of *KLK2*, *KLK3*, *TMPRSS2* mRNA by ~30% to ~70% at 16 and 24 h induction, and *FKBP5* by 50% at 16 h (Fig. 3c–f). However, over-expression of *MIR222HG* did not significantly affect the expression of AR-regulated *PLZF* and *cdc20* (Figs. 3g, h). Meanwhile, control transfections with an empty vector did not affect the response of LNCaP to DHT and over-expression of either the *MIR222HG* isoforms had no significant impact on AR expression in LNCaP (Fig. 3i). These results suggested that expression of *MIR222HGs* can significantly affect AR signaling leading to altered expression of a subset of AR-regulated genes in PCa cells. In sum, two types of ncRNAs, lncRNA *MIR222HGs* and *miR-221/222*, are transcribed from the 23.3 kb *miR-221/222* locus driven by a single differentially activated promoter in LNCaP-Abl and expression of *MIR222HGs* and/or *miR-221/222* can independently promote the CRPC phenotype.

We further investigated whether *MIR222HGs* might be directly involved in the AR complex, thus affecting AR signaling. We found no association of *MIR222HGs* with the AR complex by RNA-IP pull-down assays in *MIR222HG*-over-expressing LNCaP cells under DHT treatment (Fig. S3). Additional ChIP assays comparing the AR occupancy at the *KLK3* and *KLK2* promoter regulatory regions indicated that AR binding to androgen-responsive elements at the *KLK3* or *KLK2* locus was not affected by the expression of *MIR222HGs* in LNCaP (Fig. S4). Furthermore, the efficiency of AR translocation into the nucleus with DHT treatment was not affected in *MIR222HG*-over-expressing LNCaP cells compared to control cells (Fig. S5). These data suggested that the *MIR222HG* over-expression induced down-regulation of some AR-controlled genes most likely is not due to a direct effect on AR, but may be ascribed to its effect on factors associated with the AR machinery, a hypothesis which remains to be investigated.

### *MIR222HGs* are associated with the Argonaute complex

*MIR222HG*-332 and *MIR222HG*-464 contain regions of strong stable hairpin stem loops (Fig. 2) and potentially function as regulatory RNAs. In view of the structure of *MIR222HGs* and the biological–functional similarity between *MIR222HGs* and *miR-221/222*, we postulated the possibility that *MIR222HGs* may be functionally associated with miR-mediated processes and thus explored the potential association of *MIR222HG*s with the machinery related to the biogenesis and function of miR.

**Fig. 4:**
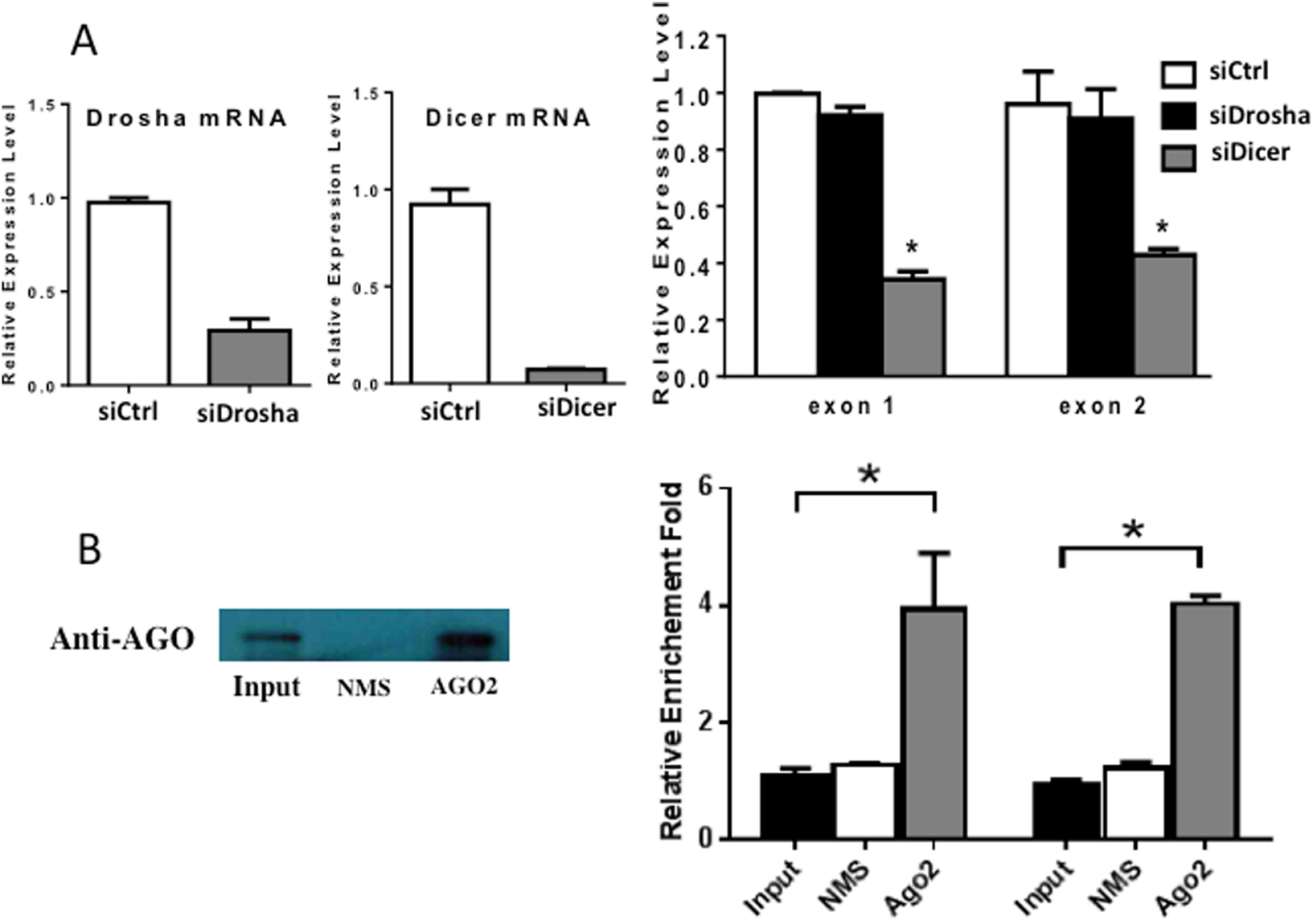
Relationship of *MIR222HGs with* Drosha, Dicer, and Argonaute 2 (AGO2). **a** The impact of knocking down Drosha or Dicer 1 on the expression level of *MIR222HGs*. LNCaP-Abl was transfected with siDrosha, siDicer 1, and siCtrl (siControl), separately. Total RNAs were isolated post-transfection and RT-PCRs were performed to determine the mRNA levels of Drosha and Dicer 1 in the left panel and the level of *MIR222HGs* as measured by primers specific for exon 1 and exon 2, in the right panel. **b** The association of *MIR222HGs* with AGO2. Total cell lysates of LNCaP-Abl were IPed with non-immune IgG and anti-AGO2 antibody, respectively. The Ago 2 protein level in the input and IP pull-down materials were measured by western blot as shown in the left panel. The amount of *MIR222HGs* in IP pull-down materials was analyzed by RT-PCR using probes specific for exon 1 and exon 2, respectively, as shown in the right-hand panel

We first determined the impact of knocking down *Drosha* and *Dicer 1* on the expression level of *MIR222HGs* in LNCaP-Abl. Drosha is a member of ribonuclease III and responsible for the initial step of miR-precursor processing in the nucleus. Dicer 1, a member of RNaseIII family, cleaves double-stranded RNA and pre-miRs into small double-stranded RNA fragments (small interfering RNA (siRNA)) and miRs in the cytoplasm. Following cleavage, Dicer docks the matured miR or siRNA onto the RNA-induced silencing complex (RISC), facilitating the function of the RISC catalytic component, Argonaute (AGO), for RNA interference. Knocking down Drosha did not significantly affect the expression level of *MIR222HGs*, *as anticipated*, as shown by the transcript level of exon 1 and exon 2 (Fig. 4a), since *MIR222HG* is not an miR. Surprisingly, knocking down Dicer 1 drastically reduced the level of *MIR222HG* (to <40%; Fig. 4a), suggesting that *MIR222HGs*’ maturation may occur via Dicer 1-associated machinery. We further performed an AGO pull-down assay to determine whether *MIR222HGs* are associated with AGO complex, since Dicer docks its processed miRs onto the AGO-associated RISC. As shown in Fig. 4b, the level of *MIR222HG* is significantly enriched in the anti-AGO2 pull-down material (indicated by exon 1 and exon 2), compared to those in the input material and in the material pull-down by the non-immune IgG, indicating the close association of *MIR222HG* with the AGO2 complex. The fact that *MIR222HG* is AGO-bound and its expression level is Dicer-dependent suggested that *MIR222HGs* may be functionally involved in the efficiency of miR-mediated RNA interference.

### The association of a high-level expression of *MIR222HGs* with the human CRPC

**Fig. 5:**
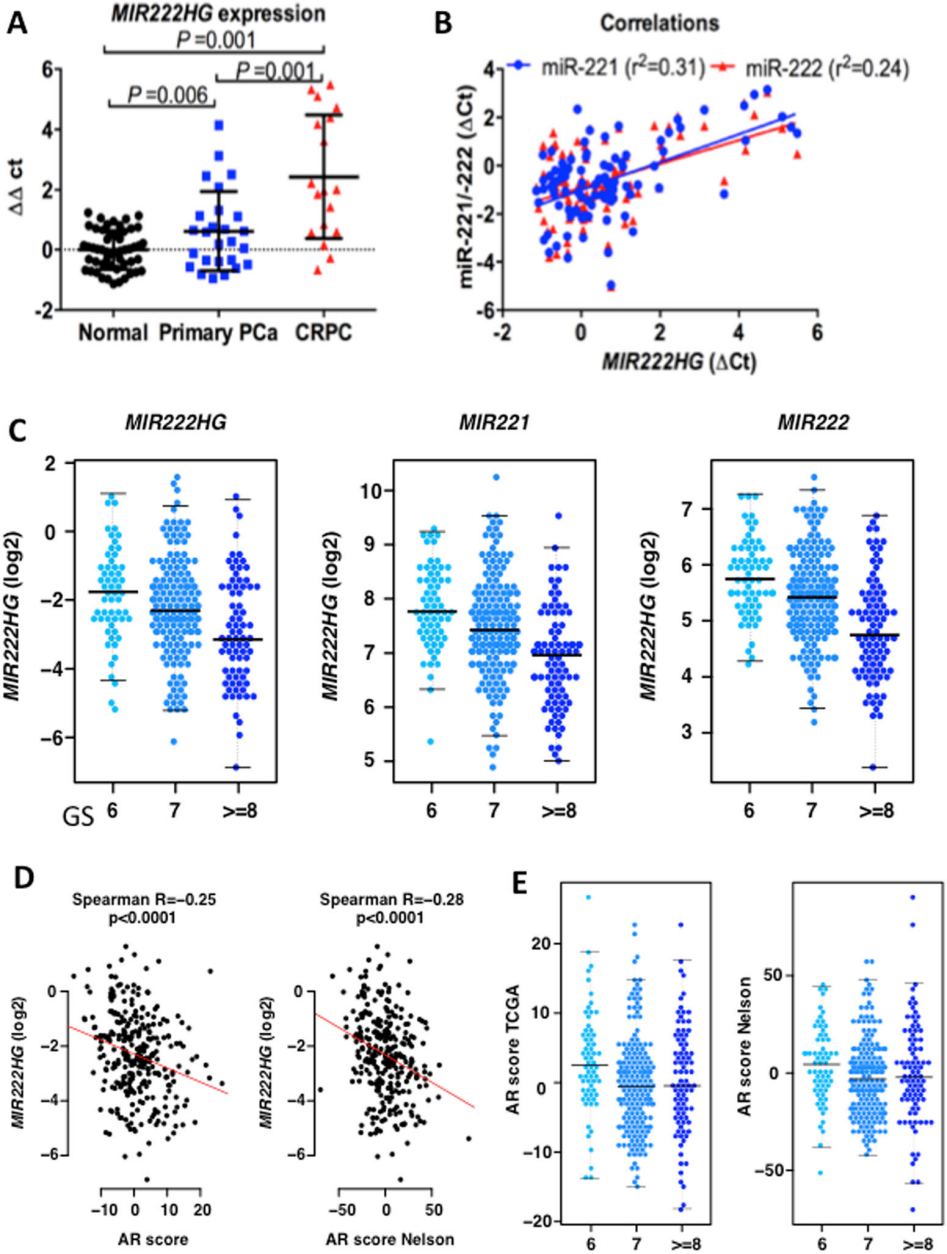
*MIR222HG* expression pattern in normal prostate, primary tumors, and CRPC. **a** Comparison of the *MIR222HG* expression. Expression levels of *MIR222HG* were measured by quantitative RT-PCR on 86 normal prostate tissues (black dots), 34 hormone-sensitive primary prostate tumors (blue squares), and 17 metastatic CRPC tissues (red triangles). All expression levels were normalized by 28S ribosome RNA (RPS28). Values represent in qPCR cycle differences relative to the mean expression level of normal tissue, which was set as 0. *P* values were estimated by one-way ANOVA. **b** The expression correlation between *MIR222HG* and *miR-221* or *miR-222* in human prostate tissue specimen. The Pearson's correlation coefficient indexes (*r*^2^) were calculated by comparing normalized qPCR cycle values (∆cts) in detecting *miR-221* (blue) or *miR-222* (red) with ∆ct value of *MIR222HG*. **c** Association of expression levels of MIR222HG, miR-221, and miR-222 with GS in primary tumors of the TCGA data set (*p* < 0.0001 Kruskal–Wallis test). **d** Correlation of *MIR222HG* expression level with AR activity (indicated by AR score) in primary tumors of the TCGA data set. AR activity score was inferred by the induction of AR target genes^24,25^. AR score Nelson indicates that the AR score was re-evaluated using the AR gene index previously published by Dr. Nelson’s laboratory^25^. **e** Correlation of GS with AR activity score

To determine the clinical significance of *MIR222HG* expression, we analyzed the expression levels of the *MIR222HG* in 86 normal prostate tissues, 34 localized HSPC primary tumors, and 17 bone metastatic CRPC samples (Fig. 5a). The expression of *MIR222HGs* was significantly higher in CRPC specimens compared to those in HSPC and normal prostate tissues 2.4-fold and 4.4-fold, respectively (both *p* < 0.001, Fig. 5a). The expression level of *MIR222HG* in HSPC tumors was 1.8-fold higher (*p* < 0.01) than those in normal prostate tissues. Interestingly, previously we found that the expression of miR-221/222 was significantly down-regulated in HSPC primary tumors compared to normal prostate tissues^23^. We further performed a Pearson's correlation analysis and found that *MIR222HG* expression only modestly correlated with expression levels of miR-221/222 (Fig. 5b).

Upon further analysis of the expression data from the TCGA data set, we found that the expression of *MIR222HG* and *miR-221/222* were inversely correlated with Gleason score (GS) in HSPC primary tumors (*p* < 0.0001 Kruskal–Wallis test; Fig. 5c). Interestingly, we also observed an inverse correlation of *MIR222HG* expression with AR transcriptional activity, as inferred by the induction of AR target genes and this result was confirmed using two AR scores (Fig. 5d)^24,25^. A higher AR score was significantly associated with Gleason 6 (Fig. 5e). Additionally, the expression of *MIR222HG* and *miR-221*/*222* significantly correlated with the degree of copy number alterations in primary PCa (Fig. S7)^24^. Expression of *MIR222HG* and *miR-221*/*222* appears to be inversely correlated with overall copy number burden (fraction of genome altered). Moreover, primary tumors that exhibit the highest burden of copy number alterations (more SCNA cluster)^24^ show a significantly lower expression (*p* < 0.0001) of *MIR222HG*, *miR-221*, and *miR-222* compared to tumors with the lowest copy number burden (Quiet cluster)^24^. Taken together, in human PCa, *MIR222HG* expression is correlated with prostate tumor progression and CRPC development, and was in part independent of miR-221/222 expression.

## Discussion

Recent advances in genomics revealed that >90% of human genome is transcribed and only a small portion of the genome is translated into proteins, and the remaining is largely transcribed as ncRNAs including miRs and lncRNAs^7^. An abundance of evidence demonstrated that the expression alteration of specific miRs or lncRNAs significantly affect cell developmental processes or cancer development and progression^26^. However, very little is known about mechanisms controlling the differential regulation of most miRs or lncRNAs at different developmental stages in normal or tumor cells.

**Fig. 6:**
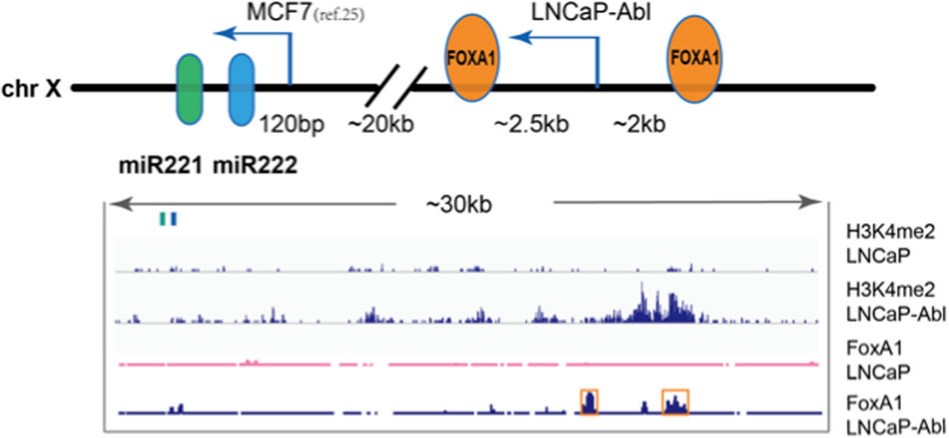
A graphical representation of the *miR-221/222* locus. Upper panel demonstrates a schematic diagram of *miR-221/222* genomic loci. The proximal promoter region was initially identified in MCF7 and the distal promoter region was identified in LNCaP-Abl as described in Fig. 1. Lower panel demonstrates the ChIP-seq enrichment of H3K4Me2 and FoxA1 binding sites in LNCaP and LNCaP-Abl cells. Each data track shown is on the same scale for both LNCaP and LNCaP-Abl cells

We present here a detailed analysis of the *miR-221/222* gene locus. *MiR-221 and miR-222* are tandemly linked on the X chromosome and processed from a single pri-miR. *MiR-221/222* are highly expressed in CRPC and their expression promotes CRPC development^16,17,23^. In examining mechanisms accounting for the differential activation of *miR-221/222* in PCa, especially during the CRPC development, we identified novel lncRNA *MIR222HGs*, derived from the immediate downstream region of a differentially activated promoter located ~23.3 kb upstream of the *miR-221/222* gene. Activation of this remote promoter in CRPC cells leads to a high production of *miR-221/222* and *MIR222HGs*. Our data suggested that *miR-221/222* and *MIR222HGs* are most likely transcribed from a single promoter and originate from the same large primary transcript in LNCaP-Abl. Apparently, in the MCF7 cell line, transcription of *miR-221/222* starts at ~120 bp upstream of *miR-222* and the minimal-promoter region was mapped to a −150 to −50 bp region^27^. In melanoma cells, the *mir-221/222* promoter was mapped to within 500 bp upstream of the *miR-221/222* gene and could be repressed by PLZF^28^. We referred to the promoters immediately adjacent to the 5′ end of the *miR-221/222* gene as proximal promoters (Fig. 6). Our histone methylation data did not identify the proximal promoters in the *miR-221/222* locus in LNCaP and LNCaP-Abl cell lines. In contrast, in LNCaP-Abl, but not in LNCaP, a putative transcription start site was found ~23 kb upstream of *miR-221/222* gene and we referred to this putative promoter region as a distal promoter (Fig. 6). We hypothesize that the proximal promoters may drive the basal level transcription of *miR-221/222* in HSPC cells, while the distal promoter functions in the transcriptional activation of *miR-221/222* in LNCaP-Abl CRPC cells. In searching for transcription factors potentially regulating the distal promoter activity, we identified two putative FoxA1 binding sites located ~5 kb region around the distal promoter in LNCaP-Abl using the previously published ChIP-seq data^18^. Simply knocking down FoxA1 yielded a mild reduction of the RNA level of *MIR222HG* and *miR-221/222* (Fig. S8), suggesting the potential involvement of FoxA1 in the differential transcription of the *miR-221/222* locus. However, this result remains to be further confirmed by quantitative FOXA1 ChIP analysis.

As mentioned, we identified two lncRNA *MIR222HG* isoforms of 332 and 464 bp, co-transcribed with *miR-221/222* from the distal promoter. Since the entire 23 kb could be transcribed as indicated by the ENCODE data, it is not excluded that other lncRNAs may also be generated from the large primary transcripts via processing events. It is possible that we may have missed some of them due to their low abundance and the abundance of different lncRNAs may vary in different types of cells. Apparently, an angiotensin II-up-regulated lncRNA, *Lnc-Ang362* of 1758 bp, is also derived from the *miR-221/222* distal promoter region in vascular smooth muscle cells (VSMCs)^29^. Knocking down *Lnc-Ang362* reduced the expression of the co-transcribed *miR-221/222* and *MCM7* in VSMCs, leading to reduced cell proliferation^29^. However, we did not find that over-expression or knockdown of the *MIR222HG*-332 bp and *MIR222HG*-464 bp had a significant impact on the expression level of MCM7 in LNCaP and LNCaP-Abl (Fig. S6)

The most surprising observation is that expression of lncRNA *MIR222HG* itself was significantly associated with the development of CRPC phenotype in PCa cell lines and tumor tissues. Over-expression of *MIR222HG* significantly decreased transcription of some DHT-induced AR-mediated genes and promoted androgen-independent growth of LNCaP. CRPC specimens had higher *MIR222HG* lncRNA expression than did primary PCa tissue. Interestingly, increased expression level of *MIR222HG* is associated with lower GS and decreased AR activity score in the primary tumors of the TCGA data set. Assuming that tumors with high AR activity and low Gleason grade respond better to ADT as suggested by the association of GS with time to progression on ADT^30^, then it is possible that tumors with low AR activity/high GS and high *MIR222HG* may be more resistant to ADT. This scenario is consistent with the finding of a higher level expression of *MIR222HG* in bone-derived mCRPC than that in HSPC localized tumors^23^.

As opposed to miRs, lncRNAs can regulate their target genes through diverse mechanisms. Our data demonstrate that the impact of *MIR222HG* expression on promoting the CRPC phenotype was likely not due to a direct association with AR, but may have resulted from the alteration of factors associated with the AR machinery, a hypothesis remained to be confirmed. The facts that *MIR222HGs*-332/464 are AGO-associated and their expression in LNCaP-Abl is Dicer 1-dependent suggested the possibility of the functional involvement of *MIR222HG-*332/464 in the AGO-associated RISC, which may in turn lead to modulation of AR signaling. As reported that *miR-221/-222* promotes androgen independence in PCa cells in part due to down-regulation of HECTD2, a direct target of *miR-221/222*^17^. HECTD2 is an essential E3 ligase involved in the degradation of PIAS1, which is a potent anti-inflammatory protein and mediates the cross-talk between nuclear factor-κB and AR signaling^31,32^. We are currently investigating the potential involvement of *MIR222HGs* in miR function and the role of Dicer 1 in the biogenesis of *MIR222HGs*.

In summary, the *miR-221/222* locus extends ~23 kb, activation of the distal promoter leads to the transcription of large primary transcripts in CRPC cells, which are further processed to generate at least two different types of ncRNAs, the miR-221/222 and the lncRNA *MIR222HGs*. Both the expression of miR-221/222 and *MIR222HGs* can significantly promote the CRPC phenotype. Further studies are needed to address the specific role of this newly identified lncRNA *MIR222HGs* in AR-mediated transcription regulation and CRPC development. Nevertheless, this study reveals a new potential function of lncRNAs as a modulator of AGO-mediated RISC.

## Materials and methods

### Cell culture

LNCaP was obtained from ATCC and maintained in RPMI-1640 with 10% FBS. LNCaP-Abl was provided by Zoran Culig (Innsbruck Medical University, Austria)^33^ and maintained in RPMI-1640 with 10% CFBS^16^. All cells were regularly screened for mycoplasma using a Venor GeM Mycoplasma Detection Kit (Sigma), passaged for fewer than 4 months and used at early passages (<20).

### Quantitative RT-PCR

For *MIR222HG* expression, strand-specific cDNAs were generated by RT and then subjected to quantitative RT-PCR (qRT-PCR) analysis by FastStart Universal SYBR Green Master (Roche, IN, USA). *MiR-221/222* levels were quantitated by qRT-PCR, using TaqMan miR assays from Applied Biosystem^16^. Real-time PCR primers/probe sets for *KLK3*, *KLK2*, *TMPRSS2*, *FKBP5*, *AR*, *Drosha*, *Dicer 1*, and *GAPDH* were inventoried products of Applied Biosystem.

### Northern blot analyses

Poly(A)+ RNAs were isolated by Invitrogen Dynabeads mRNA Purification Kit (Carlsbad, CA, USA). About 100 ng poly(A)+ RNA was fractionated in 1.5% (w/v) denaturing agarose gel and transferred onto nylon membrane, which was subsequently hybridized with digoxigenin (DIG)-labeled DNA probes and detected by DIG Detection Kit (Roche, IN, USA).

### Rapid amplification of cDNA ends

5′RACE and 3′RACE were performed using Invitrogen Kits (Carlsbad, CA, USA). For 5′RACE, ~10 μg of total RNA from LNCaP-Abl was sequentially treated with Calf Intestine Phosphatase and Tobacco Acid Pyrophosphatase prior to ligation with adaptor RNAs. The ligation products were subjected to RT-nested PCR. For 3′RACE, ~3 μg total RNA was treated by 10 U poly(A) polymerase for 30 min at 37 °C. The poly(A)-tailed RNAs were amplified by RT-nested PCR. Primer information is in Supplementary Table 1. The RACE products were analyzed by 1% agarose gel and sequenced.

### Dual-luciferase reporter assay

The different sized DNA fragments were cloned into pGL4 promoter luciferase reporter vector (Promega, WI, USA). LNCaP-Abl was seeded at a density of 2 × 10^5^ cells per 12-well dish. Twenty-four hours later, 400 ng of each construct was co-transfected with 20 ng of pRL-TK as transfection efficiency control, using Lipofectamine 2000. Forty-eight hours after transfection, luciferase activity was measured by Dual-Luciferase Reporter Assay Kit (Promega).

### MIR222HG silencing and over-expression

Customized siRNA SmartPool targeting *MIR222HG*s was synthesized by GE Dharmacon (Lafayette, CO, USA) and their sequences were included in Supplementary Table 1. LNCaP-Abl was seeded into 6-well plates and transfected with 30 nM of SmartPool siRNA using Lipofectamine RNAiMax Reagent (Invitrogen). CDNAs of *MIR222HG*s were cloned into pcDNA3.1(+) vector (Invitrogen) and confirmed by sequencing. LNCaP was transfected with *MIR222HG*-over-expressing vectors and selected by 500 μg/mL geneticin (G418, Invitrogen).

### Cell proliferation assay

For all cell growth studies, cells were plated in 96-well plates. Twenty-four hours after seeding, cells were transfected with anti-miR-221 or *MIR222HG* siRNAs. Tetrazolium salt cell proliferation assay (WST-1, Roche Applied Science) was used to determine proliferation.

### Ago RIP-ChIP analysis

Ago RIP-ChIP was performed as previously described^17^. Briefly, 10^7^ LNCaP-Abl were lysed. Cell lysates were collected, cleared with pre-blocked protein-G beads (Invitrogen), and used for co-IP with either anti-AGO G beads, NMS G beads (Pierce Biotechnology) at 4 °C for 90 min. After co-IP, the beads were washed and treated with DNase I. Co-immunoprecipitated RNAs were extracted using TRIzol (Invitrogen). The associated *MIR222HG* transcripts were measured by RT-PCR. The AGO2 (C34C6) Rabbit-mAb #2897 is from Cell Signaling Technology.

### Prostate tissue or tumor-derived RNAs

Total RNAs isolated from primary PCa tumor tissues, their adjacent normal tissues, and bone marrow biopsies were as previously described^23^.

### Bioinformatic analyses

Genome-wide histone modification data from multiple cell lines was derived from ENCODE HG19 data^19^. The ChIP-seq data sets of LNCaP and LNCaP-Abl were as previously described^18,34^. ORF Finder from NCBI (http://www.ncbi.nlm.nih.gov/gorf/gorf.html) and CPC (http://cpc.cbi.pku.edu.cn)^21^ were used to define the non-coding potential. RNA structure was predicted using RNAfold from the Vienna RNA package^22^.

### Statistical analysis

A two-tailed *t* test was used for statistical *p* value analysis. Error bars represent standard deviation of three independent experiments. The Pearson's correlation coefficient was used to determine the expression correlation between *miR-221/222* and *MIR222HGs* in tumors tissues. Spearman's correlation coefficient was used to determine the correlation among *miR-221/222* and *MIR222HG* expression in the TCGA data set. The significance of expression association of *MIR222HG*, *miR-221*, *miR-222*, and GS in the TCGA data set was evaluated using Kruskal–Wallis test. All plots and statistical analysis were performed in R (version 3.3.1). Fraction of genome altered and copy number clustering data are available at http://www.cbioportal.org/.

## Electronic supplementary material

Additional experimental data for the function of MIR222HGs in prostate cancer cells(DOCX 4973 kb)
