# Supplementary material for: Expression of lncRNA MIR222HG co-transcribed from the miR-221/222 gene promoter facilitates the development of castration-resistant prostate cancer
Source: Oncogenesis. 2018 Mar 13;7(3):30. doi: 10.1038/s41389-018-0039-5 (PMC5852960; doi:10.1038/s41389-018-0039-5)
Supplement: Supplementary file 1 — Additional experimental data for the function of MIR222HGs in prostate cancer cells(DOCX 4973 kb) [file 41389_2018_39_MOESM1_ESM.docx]

**Supplementary Information**

**Chromatin immunoprecipitation (ChIP) assay**

ChIP experiments were performed as previously described by Hsieh CL, *et al*. 2014. Briefly, 10 million cells were used per immunoprecipitation (IP). Cells were fixed with 1% formaldehyde solution. DNA was sonicated and subjected to IP with antibodies against androgen receptor (N-20X from Santa Cruz) or nonspecific IgG. DNA was purified and analyzed by qPCR with primer sequences as previously described.

Hsieh CL*, et al.* (2014) Enhancer RNAs participate in androgen receptor-driven looping that selectivelyenhances gene activation. *Proceedings of the National Academy of Sciences of the United States of America* 111(20):7319-7324.

**Cell fractionation and Immunoblotting**

Cells were collected and lysed with lysis buffer (50mM Tris pH8.0, 1mM EDTA, 1mM MgCl2, 150mM NaCl, 1% NP-40, 1 mM β-glycerophosphate, 1 mM Na3VO4, 1 mM NaF, protease inhibitors) and incubated for 20 min on ice. Subsequently, the cells were homogenized with 30 strokes of Dounce homogenizer followed by addition of Triton-X (final concentration 0.1%) and centrifuged at 1,200 rpm for 10 min. The pellet, corresponding to the nuclear fraction, was washed twice with lysis buffer and lysed in radio immunoprecipitation assay (RIPA) lysis buffer with proteinase inhibitor cocktail (Roche), and sonicated using BioruptorStandard® for 5 min. The supernatant, corresponding to the cytoplasmic fraction, was further centrifuged at 13,000 rpm at 4^◦^C for 15 min. Proteins were subjected on 4-15 % SDS-polyacrylamide gels before being transferred onto polyvinylidene difluoride membrane (Millipore). Immunoblotting of the membranes was performed using the following primary antibodies: anti-AR (1:2000; santa cruze), anti-H3 (1:2000; Abcam), anti-β-actin (1:5000; sigma), anti-PCNA (1:2000; abcam), or anti-tubulin (1:3000; sigma). Signals were revealed after incubation with recommended secondary antibody coupled to peroxidase by using enhanced chemiluminescence.

**RNA immunoprecipitation (RIP) assay**

RIP experiments were performed as described previously ([1](#_ENREF_1)). Briefly, 10 million cells were used per IP. Cells were fixed in 0.3% formaldehyde solution and lysed in RIPA lysis buffer with proteinase inhibitor cocktail (Roche). Total chromatin and RNAs were sonicated and subjected to IP with the same AR antibodies used in ChIP experiments or nonspecific IgG. Immunoprecipitated RNAs were isolated using TRIzol LS reagent, followed by cDNA synthesis. The *MIR222HG Detection Primers* provided in Supplementary Tab. 1 were used for qPCR.

**Suppl. Tab. 1 Utilized Nucleotide Oligos**

| *MIR222HG SmartPool siRNA* | |
| --- | --- |
| siRNA-1 | UCAAAGUGGUUGUGACCGAUU |
| siRNA-2 | GGGAACAAGACUAAACAAAUU |
| siRNA-3 | CCAGAUUGGUAAGUGAAAUU |

| *5’RACE* | |
| --- | --- |
| 5’ RACE Outer Primer-1 | GCTGATGGCGATGAATGAACACTG |
| 5’ RACE Outer Primer-2 | ATTAGGGAGCAATGCCACTTT |
| 5’ RACE Inner Primer-1 | CGCGGATCCGAACACTGCGTTTGCTGGCTTTGATG |
| 5’ RACE Inner Primer-2 | AACGTCTTTGTTCCAGATCACA |
| 5’ RACE Specific primer | TGATAGATGACCAAGTGACTGCTT |
| *3’ RACE* | |
| 3’ RACE Outer Primer-1 | GCGAGCACAGAATTAATACGACT |
| 3’ RACE Outer Primer-2 | TGATAGATGACCAAGTGACTGCTT |
| 3’ RACE Inner Primer-1 | CGCGGATCCGAATTAATACGACTCACTATAGG |
| 3’ RACE Inner Primer-2 | CAAACTCTAGGCTTCCTGAACTG |

| *MIR222HG Detection Primers* | |
| --- | --- |
| MPNR222-F | GCAGATAACTCTTTAGAA |
| MPNR222-R | TTCACTTACCAATTCTGG |

| *MIR222HG Gene Cloning Primers* | |
| --- | --- |
| MPNR222-Clone-F | CCAGTGATTCAGCCACATGT |
| MPNR222-Clone-R | CGCATATGATATCTATTAGGGAG |

| *MIR222HG Primers* | |
| --- | --- |
| Exon 1 | F: GCCACATGTGATAGATGACCA  R: CAGTTCAGGAAGCCTAGAGTTTG |
| Exon 2 | F: CCTCGTGTGATCTGGAACAA  R: ATTAGGGAGCAATGCCACTTT |

**Figure S1**

**
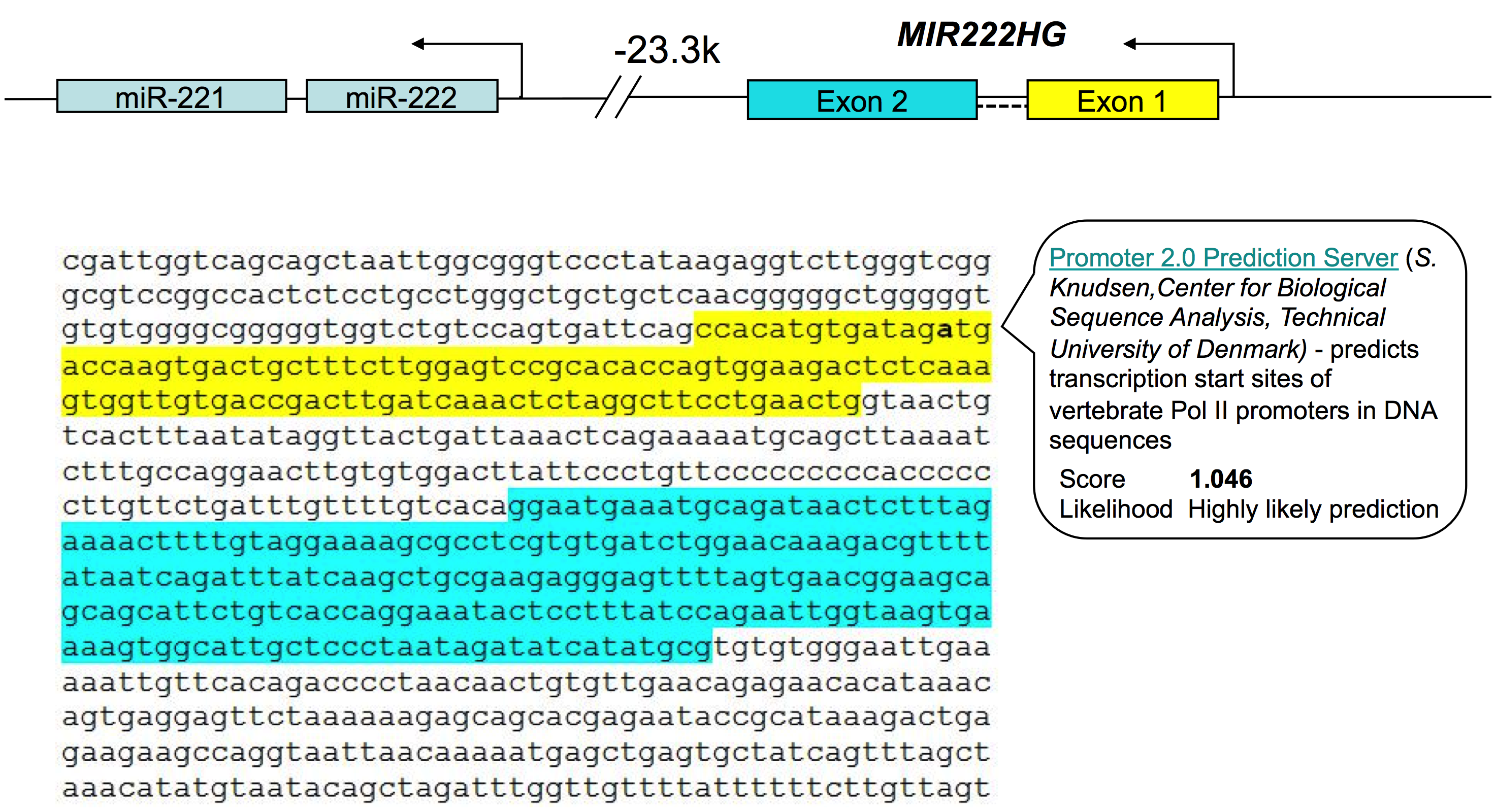
**

**Figure S1. *The schematic diagram and sequence of MIR222HG.*** Upper Panel. The schematic diagram of *MIR222HG* (the dashed line indicates a splicing option) and miR-222/-221 gene loci. Lower panel. Full sequence and predicted promoter site located in lncRNA *MIR222HG.*

**Figure S2**

**
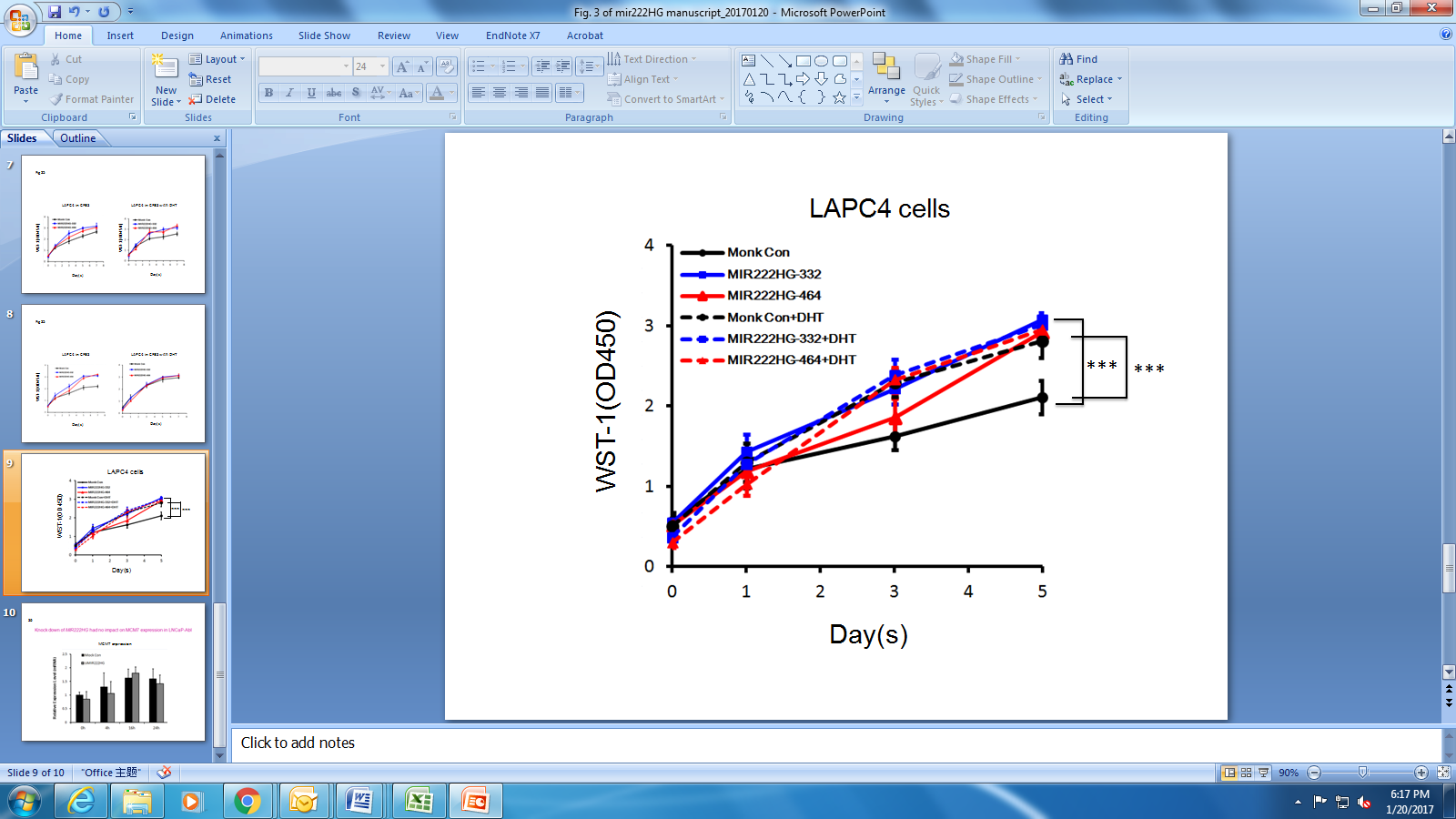
**

**Figure S2. Effect of *MIR222HG* expression level on the growth of LAPC4 cells**. WST-1 analysis of the growth of LAPC4 cells that were transfected with the empty vector (Mock Con, black lines), the *MIR222HG* 332 bp isoform expression construct (blue lines) or the *MIR222HG* 464 bp isoform expression construct (red lines) in hormone free medium (CFBS, solid lines) or CFBS supplemented with 10 nM DHT (CFBS+DHT, broken lines).

**
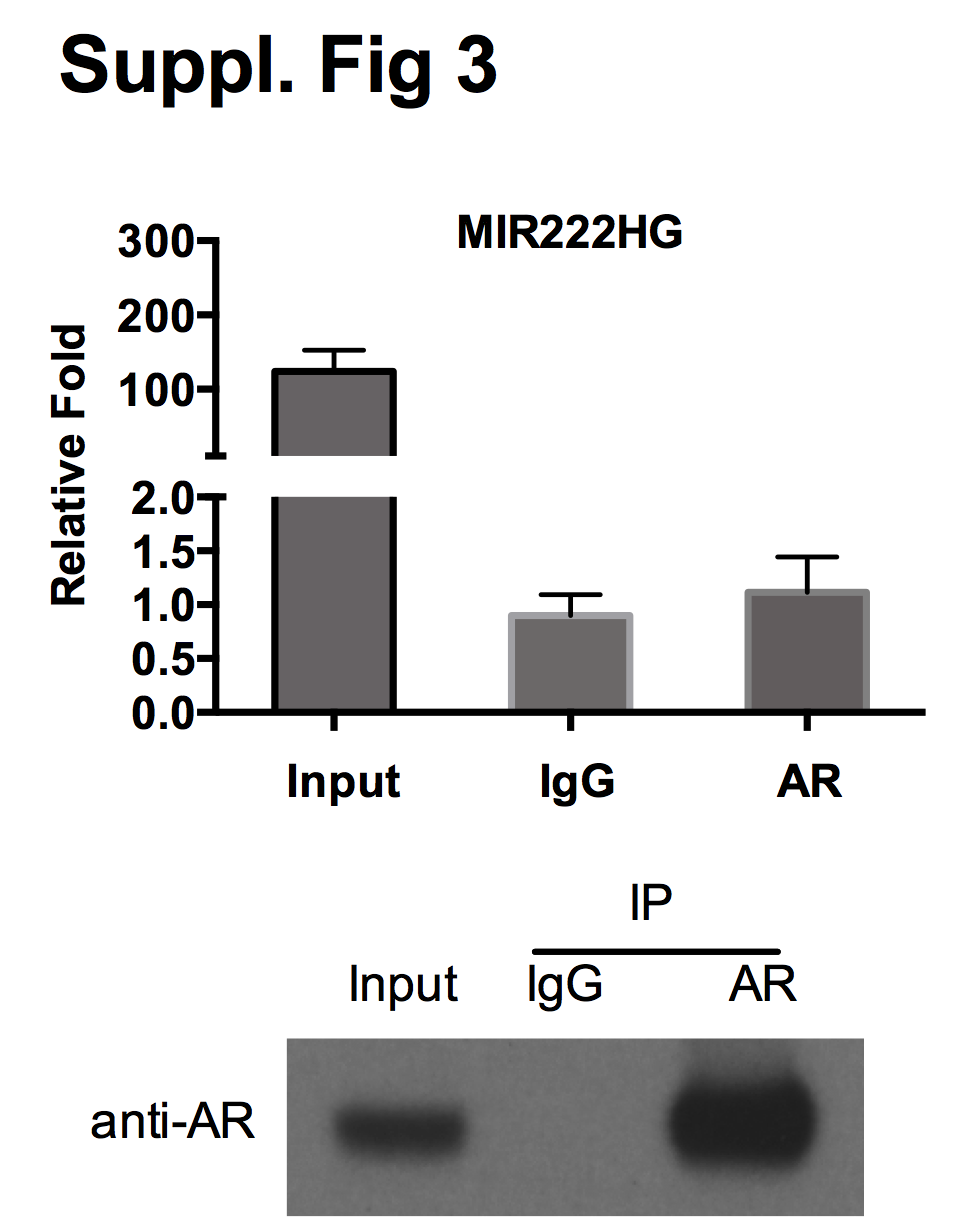
Figure S3**

**Figure S3. To examine the interaction of *MIR222HG* with AR by RIP assay.** RIP assay of AR or IgG control were performed in *MIR222HG* over-expressed LNCaP cells treated with 10 nM DHT for 6 hours.

**Figure S4**


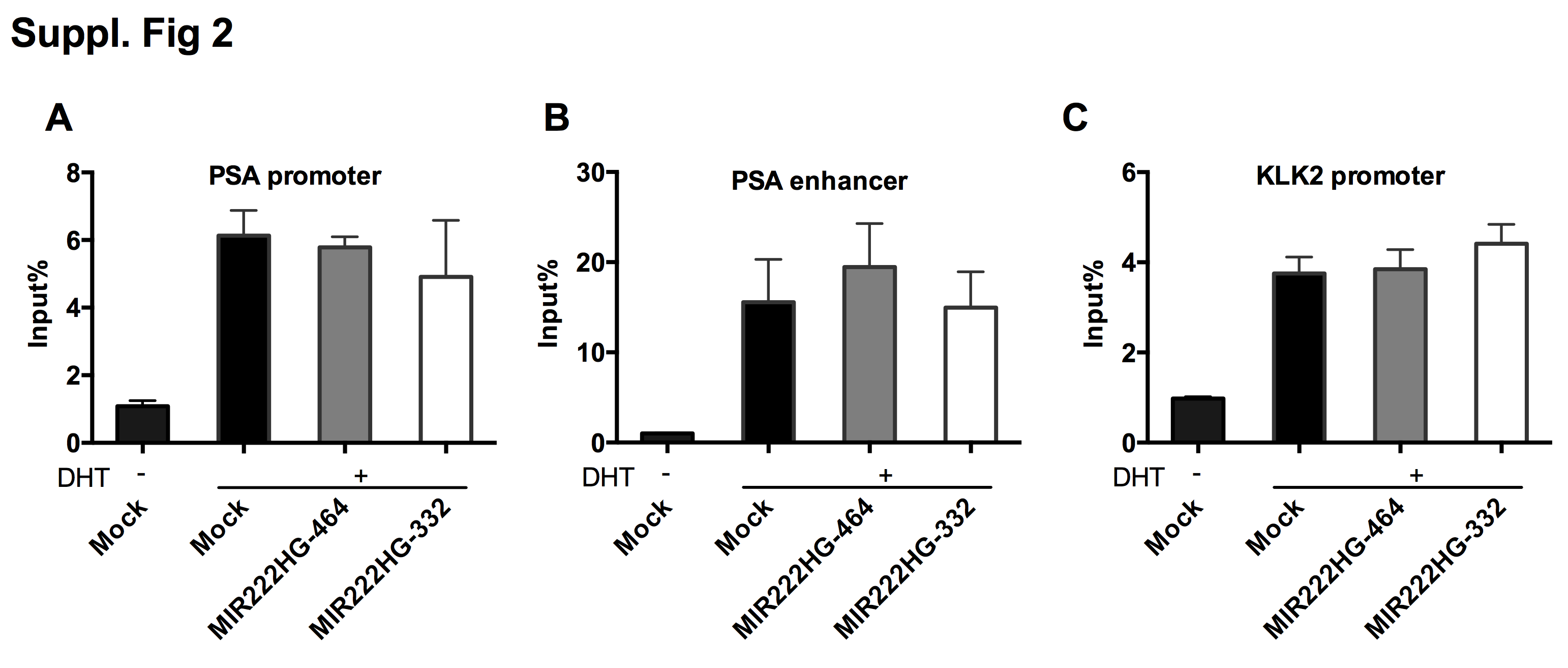


**Figure S4. The effect of *MIR222HG* expression levels on AR occupancy at the KLK3 and KLK2 promoter regulatory regions.** ChIP analyses of AR recruitment to AREs on the PSA promoter (A) or PSA enhancers (B) or KLK2 promoter (C) in LNCaP that were transfected with empty over-expressing vector, *MIR222HG* 464bp isoform or *MIR222HG* 332bp isoform. ChIP assays were performed using the anti-AR antibody in cells treated without or with DHT.

**Figure S5**

A B

**
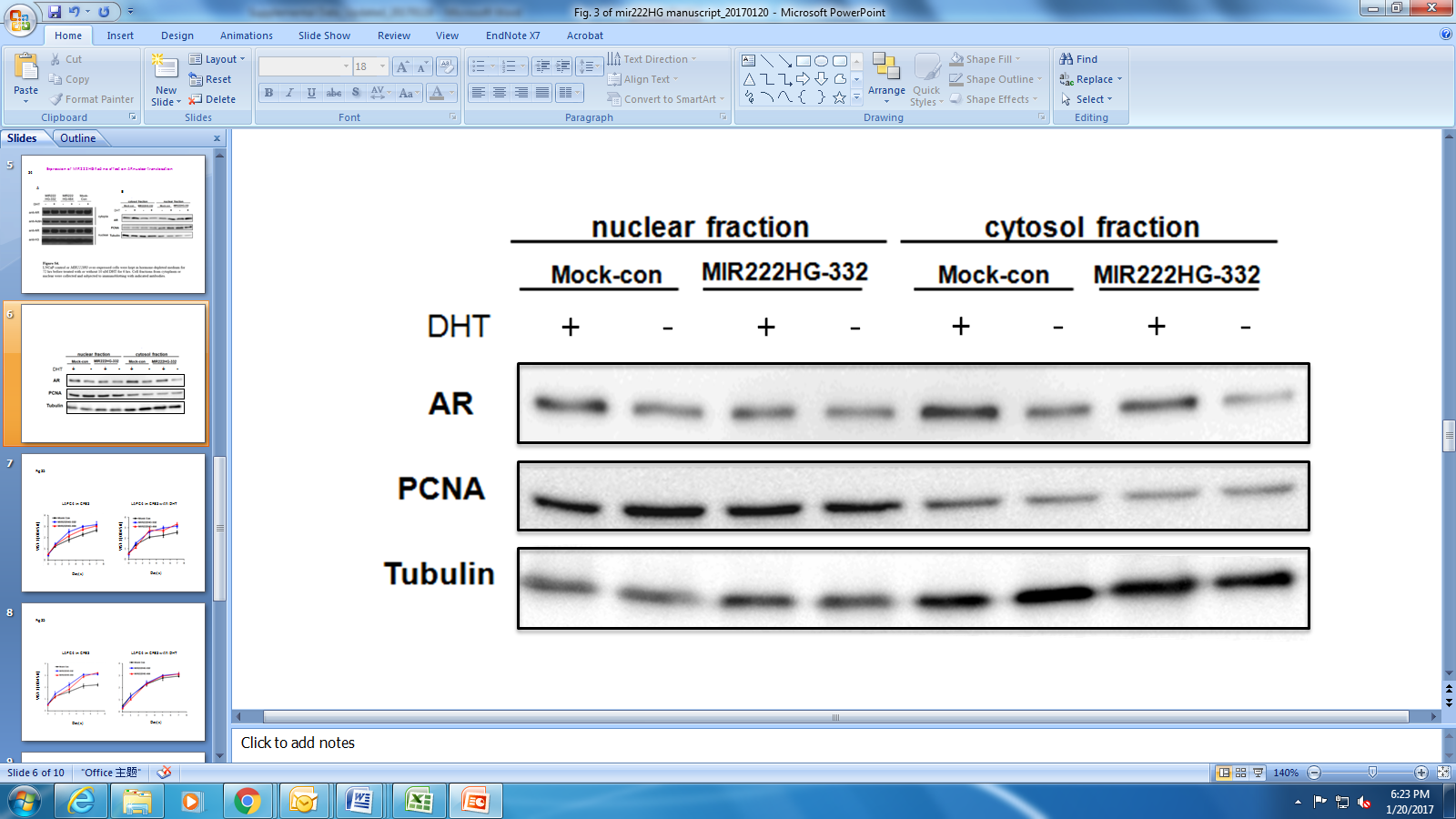
**

**
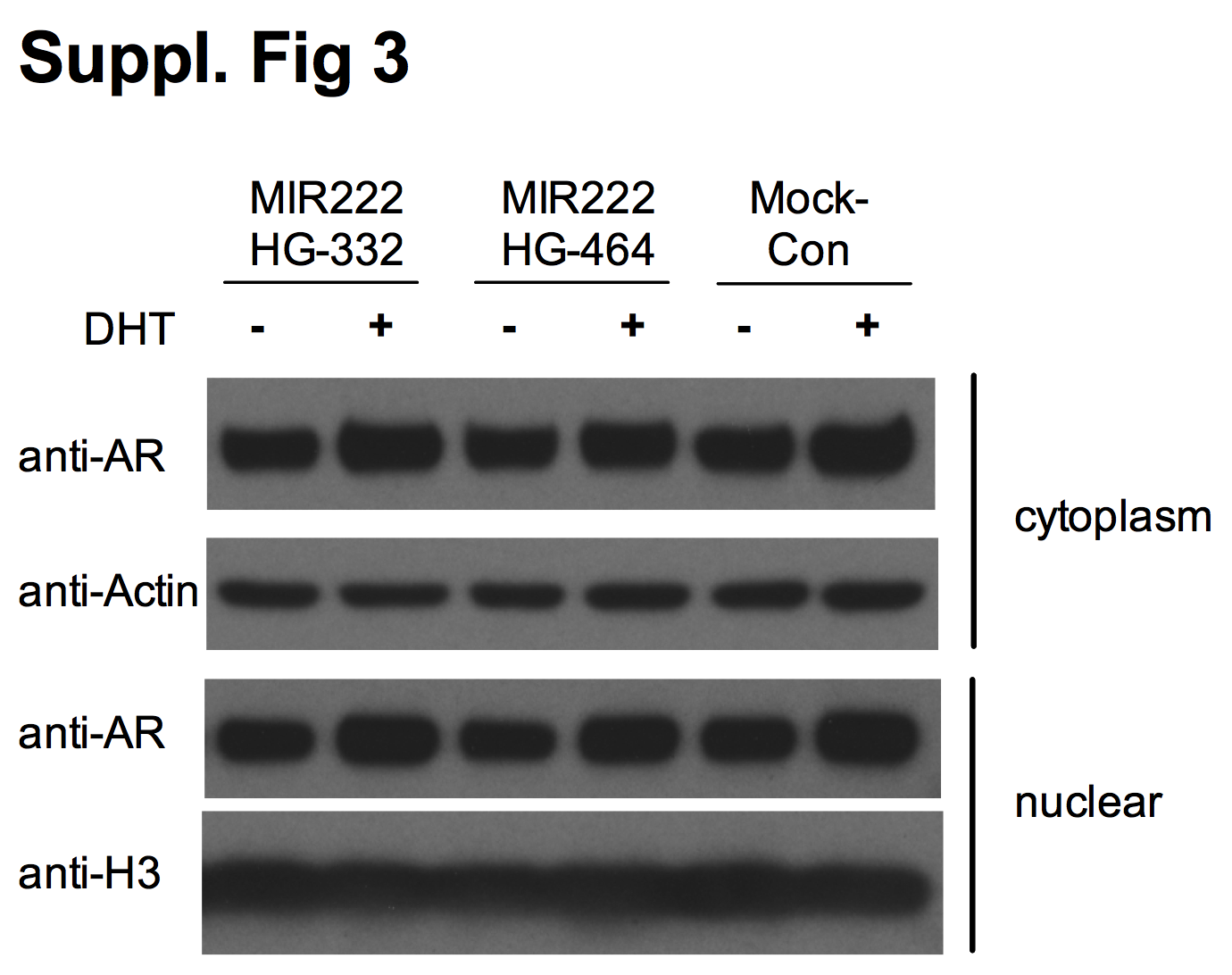
**

**Figure S5. The effect of *MIR222HG* expression levels on AR nuclear translocation.** LNCaP control or *MIR222HG* over-expressed cells were kept in hormone-depleted medium for 72 hrs before treated with or without 10 nM DHT for 6 hrs. Cell fractions from cytoplasm or nuclear were collected and subjected to immunoblotting with anti-AR (A&B), anti-H3 (A), anti-β-actin (A), anti-PCNA (B), or anti-tubulin (B) antibodies.

**Figure S6**


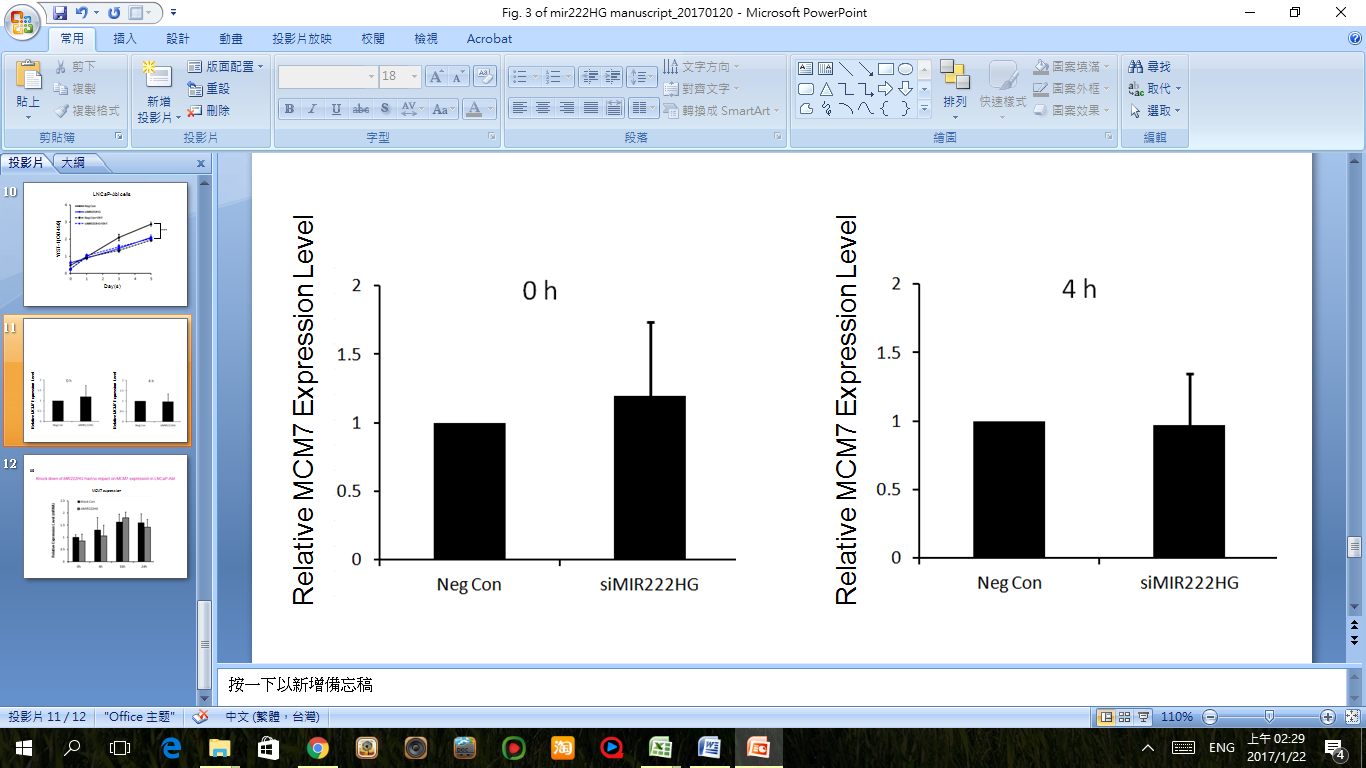


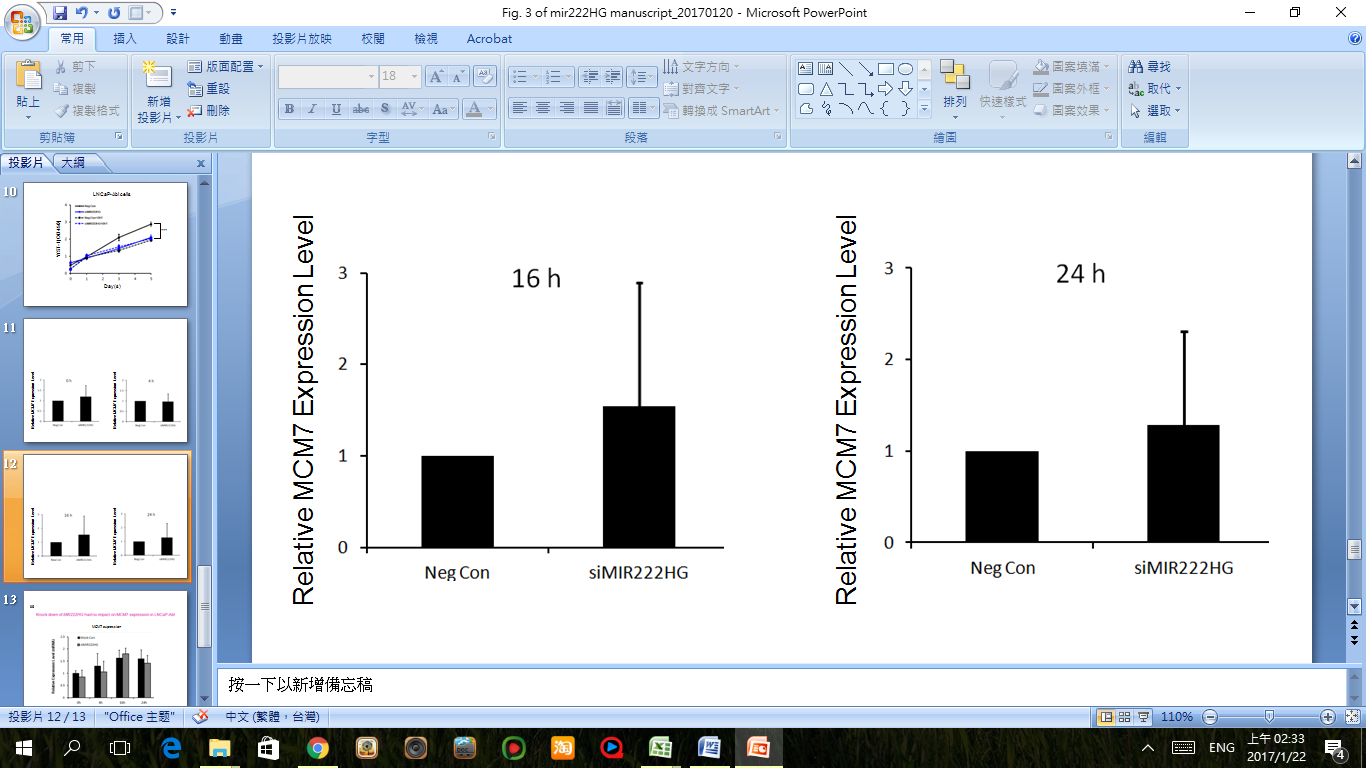


**Figure S6.** **The impact of MIR222HG expression on the mRNA expression level of MCM7 in LNCaP-Abl**. Quantitative analysis of the expression level of MCM7 in LNCaP-Abl upon DHT treatment. LNCaP-Abl cells that were transfected with the negative siRNA control (Neg Con) or *MIR222HG* siRNAs (siMIR222HG) and kept in hormone free medium (CFBS) for 0h, 4hrs, 16hrs or 24hrs. The MCM7 expression level was determined by RT-PCR. Triplicate experiments were performed for each set. The data represents mean ± S.D. (n = 3).

**Figure S7**

**
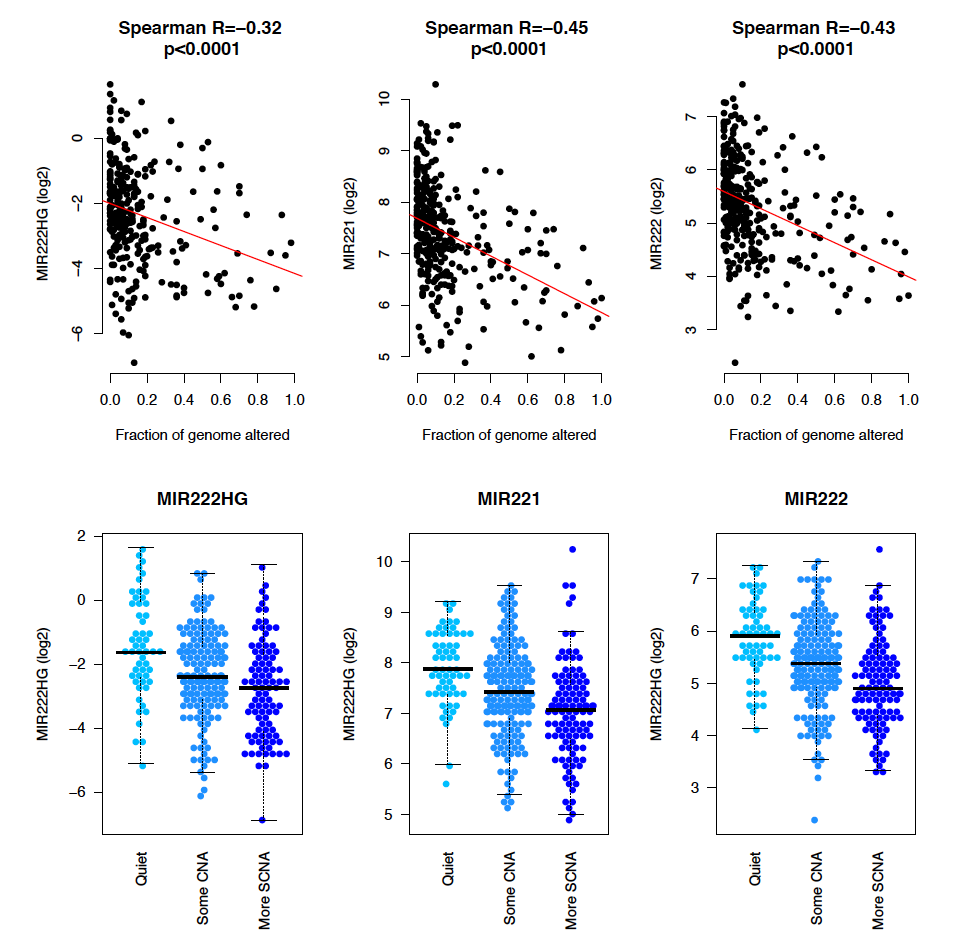
**

**Figure S7. The expression of MIR222HG, miR-221, miR-222 is correlated with the degree of copy number alterations in primary prostate cancer (TCGA, Cell 2015).**  Expression of MIR222HG, miR-221, miR-222 is inversely correlated with overall copy number burden (fraction of genome altered). Copy number clustering of primary tumors (TCGA, Cell 2015) reveals that the expression of MIR222HG, miR-221, miR-222 is significantly lower in tumors that harbor a higher extent of copy number alterations (copy number cluster= More SCNA, as indicated in TCGA, Cell 2015).

**Figure S8**


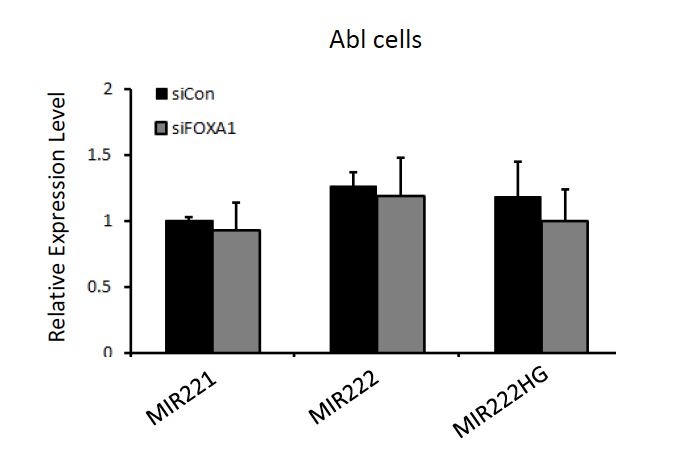


**Figure S8. The Impact of knockdown of FOXA1 on the expression level of mir221/222 and MIR222HG.** LNCaP-Abl cells were transiently transfected with negative RNAi (siCon) or FOXA1RNAi (siFOXA1), then the expression level of mir221/222 and MIR222HG were analyzed by RT-PCR.

**Figure S9**


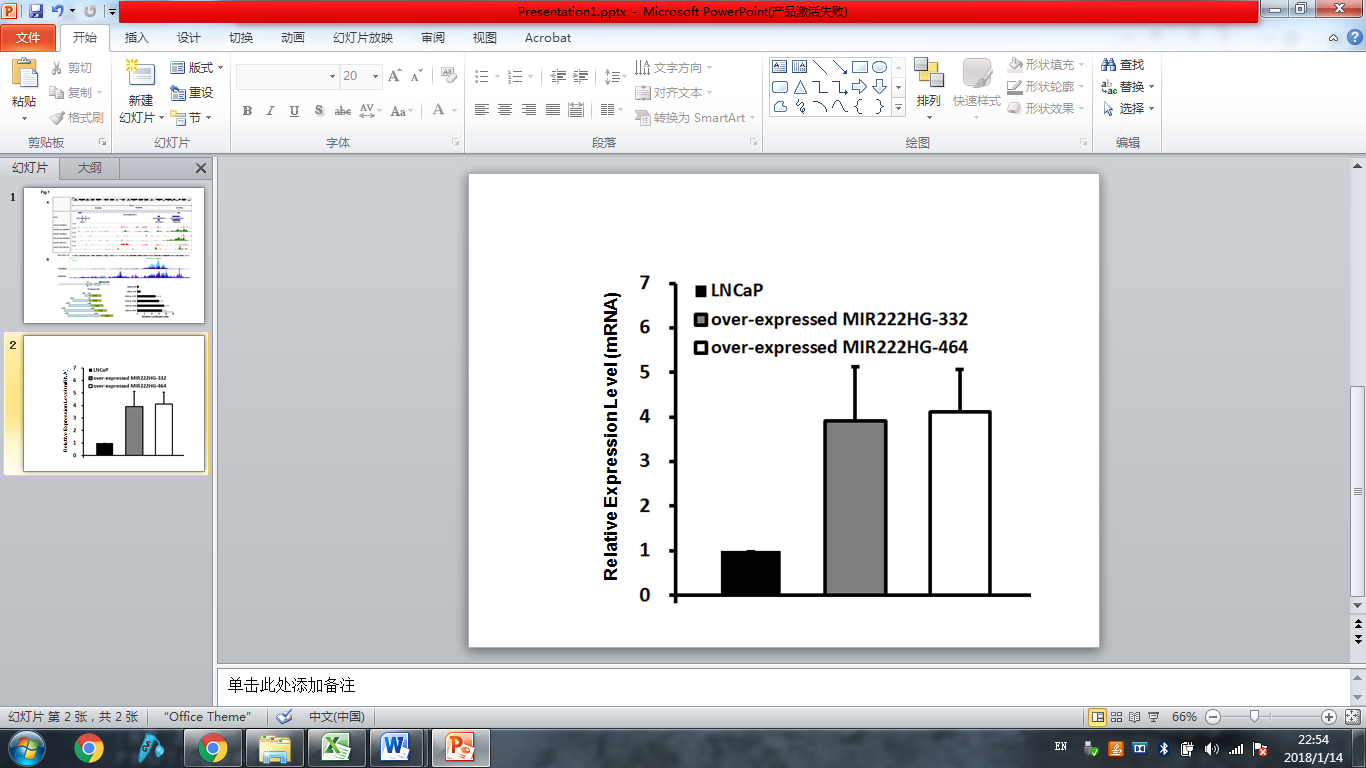


**Figure S9.** **The *MIR222HG* mRNA level in LNCaP *MIR222HG*-332 or 464 over-expressing cells**. LNCaP cells were transfected with vector only (LNCaP) or *MIR222HG* over-expressing constructs (MIR222HG-332 or MIR222HG-464). The *MIR222HG* expression level was determined by RT-PCR. Triplicate experiments were performed for each set. The data represents mean ± S.D. (n = 3).
